# Hip osteoarticular complication due to delay in diagnosis and treatment of brucellar hip arthritis: two cases report

**DOI:** 10.1186/s12879-019-4045-9

**Published:** 2019-05-14

**Authors:** Jie He, Qiang Zhang

**Affiliations:** Department of Orthopedics, Beijing Ditan Hospital, Capital Medical University, Beijing, 100015 China

**Keywords:** Brucellosis, *Brucella melitensis*, Septic osteoarthritis, Osteonecrosis, Subchondral sclerosis

## Abstract

**Background:**

Brucellosis is a systemic infectious disease frequently associated with osteoarticular involvement. While sacroiliitis is a common manifestation of brucellosis, septic osteoarthritis is less frequent. Here, we report two cases of septic osteoarthritis caused by *Brucella melitensis*.

**Case presentation:**

Both patients had a history of contact with goats before admission. Upon clinical examination, they showed marked pain and limited movement in the hip. Imaging findings revealed obvious osteonecrosis of the right femoral head. Inflammatory markers, including erythrocyte sedimentation rate and C-reactive protein level, were elevated. The tube agglutination test results of both patients were positive (1:160 and 1:200). Real-time polymerase chain reaction analysis of synovial fluid revealed the presence of *B. melitensis*. We deduced that septic osteoarthritis could explain these clinical and radiological findings. Both patients were followed-up for 12 months. They returned to their normal routine after completing a standard antibiotic regimen, including doxycycline (100 mg, daily) and rifampicin (600 mg), for 6 weeks.

**Conclusions:**

Brucellar hip arthritis is a serious clinical manifestation of brucellosis, presenting mainly as marked joint pain and limited mobility. It is characterized by joint effusion, synovitis, and soft-tissue swelling on magnetic resonance images. Physicians should consider brucellosis as one of the differential diagnoses of arthritis.

## Background

In 2010, the number of reported cases of brucellosis in China was approximately 33,000, and the incidence of brucellosis has increased even more in recent years [1]. Brucellosis is a systemic infection that can involve any organ or system of the body [2]. It affects the musculoskeletal system, most commonly the sacroiliac joints; however, this disease rarely affects the hip [3]. Brucellosis requires greater attention because of its high prevalence and high proportion of therapeutic failure [4]. Hip arthritis is a serious complication of brucellosis. It is mainly characterized by hip pain and limited mobility. In terms of clinical manifestation and imaging findings, there is no significant difference between brucellar hip arthritis and other forms of hip arthritis [5]. This often results in clinical misdiagnosis and mistreatment. Early tube agglutination test (TAT) can minimize the risk of misdiagnosis [6]. Currently, brucellosis is mainly diagnosed by polymerase chain reaction (PCR) and blood culture.

## Case presentation

### Case 1

**Fig. 1:**
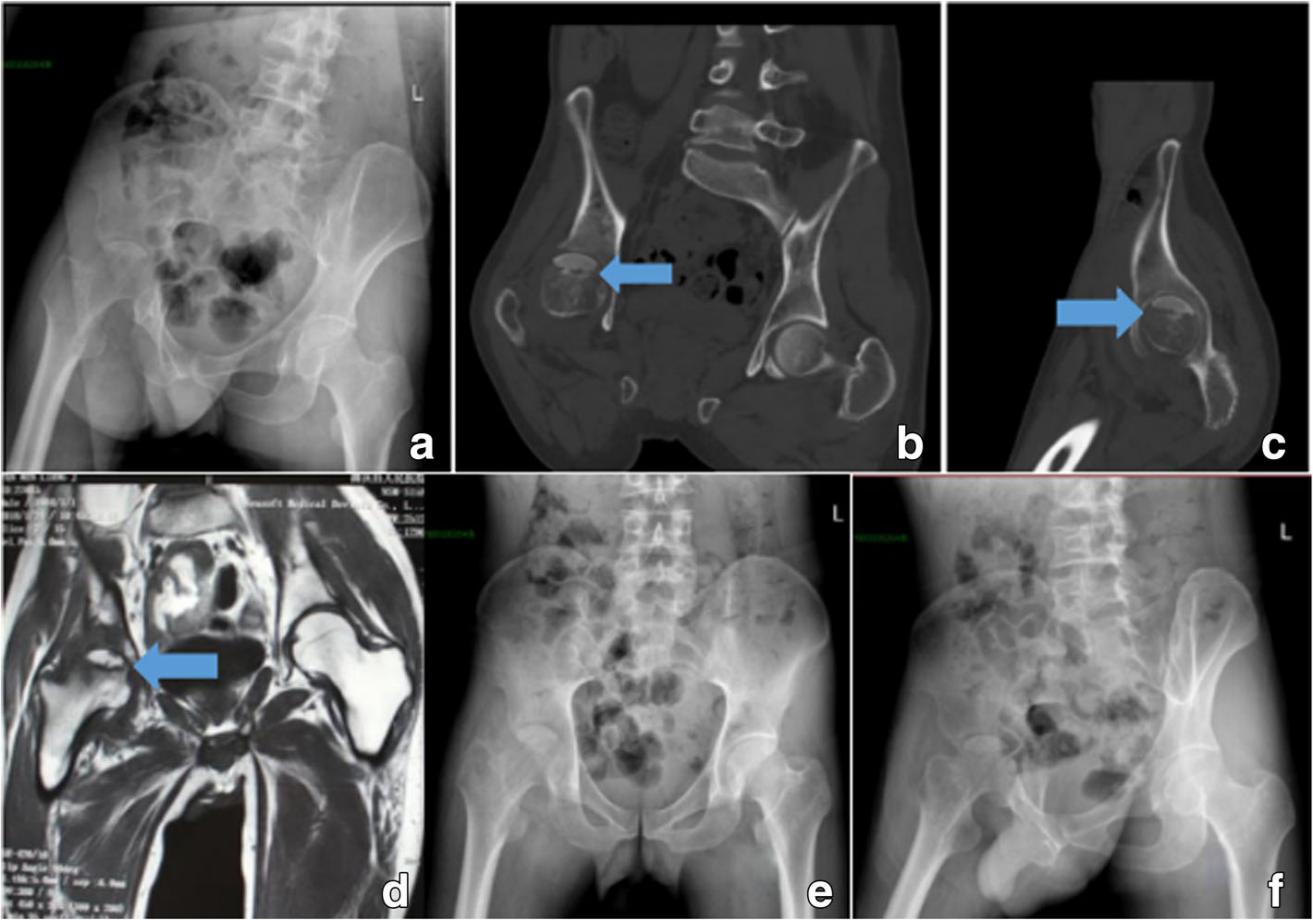
Patient 1: X-ray and computed tomography images of the pelvis showed osteonecrosis, subchondral erosion, and sclerosis (**a**–**c**). Magnetic resonance findings of the right hip (**d**) showed marked joint effusion and synovitis. Post-treatment X-ray images showed narrowing of the joint space and osteoporosis (**e**, **f**)

A 35-year-old Chinese man presented at our hospital with pain and limited movement in his right hip for the last 6 months. Although he had previously received medical treatment and joint-puncture treatment at local hospitals, there had been no improvement in his condition. His case history indicated that he had worked in wool processing 6 months before presenting at our hospital. We, therefore, suspected that the patient might have acquired *Brucella* infection through inhalation or even contact with injured skin. At the time of admission, the patient showed limited active and passive hip movement because of significant pain. His erythrocyte sedimentation rate (ESR) and C-reactive protein (CRP) level were 108 mm/h and 36 mg/L, respectively. Remarkably, his pelvic X-ray and computed tomography (CT) findings revealed osteonecrosis of the femoral head, subchondral erosion, and sclerosis (Fig. 1a–c). Magnetic resonance imaging (MRI) findings of his right hip (Fig. 1d) showed marked joint effusion and synovitis. We deduced that septic osteoarthritis could explain these clinical and radiological findings. The results of real-time PCR demonstrated the presence of *Brucella melitensis* in synovial fluid*.* During hospitalization, the patient was administered doxycycline and rifampicin for 6 weeks. By the time of discharge, his joint pain had resolved completely, and his joint mobility had increased greatly. The patient could walk normally with a walking aid.

### Case 2

**Fig. 2:**
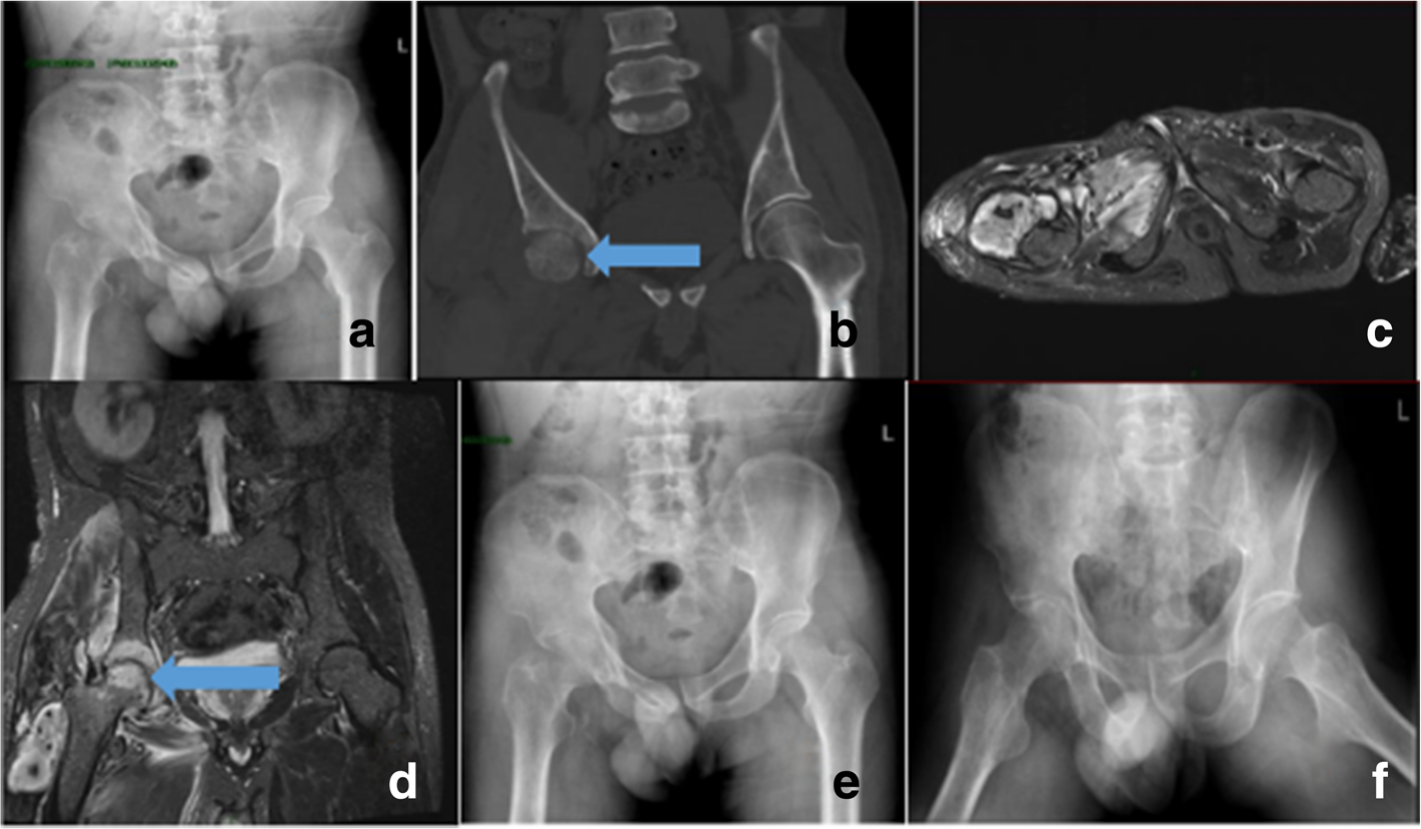
Patient 2: X-ray and computed tomography images of the pelvis showed bone destruction, subchondral erosion, and sclerosis (**a**, **b**). Magnetic resonance images of the right hip (**c**, **d**) showed marked joint effusion, synovitis, and soft-tissue swelling. Post-treatment X-ray images showed narrowing of the joint space and osteoporosis (**e**, **f**)

A 41-year-old Chinese man presented at our hospital with pain and limited movement in his right hip for the last 2 months. He had no relevant medical history or any history of injury or alcohol/steroid use. He had not received any medical treatment for his complaints. His case history indicated that he had worked in a slaughterhouse for 3 months. We, therefore, suspected that he might have acquired *Brucella* infection through ingestion or contact with injured skin. At the time of admission, the patient showed limited active and passive movement of the right hip because of significant pain. His ESR and CRP level were 80 mm/h and 29 mg/L, respectively. Pelvic X-ray and CT findings revealed bone destruction, subchondral erosion, and sclerosis (Fig. 2a–b), while MR images of his right hip (Fig. 2c–d) showed marked joint effusion, synovitis, and soft-tissue swelling. We again deduced that these clinical and radiological findings could be explained by septic osteoarthritis. The results of PCR analysis revealed the presence of *B. melitensis* in synovial fluid*.* During hospitalization, the patient was administered doxycycline and rifampicin for 6 weeks. At the time of discharge, his joint pain and swelling had resolved completely, and his joint mobility had returned to normal. The patient could walk normally without any aid.

## Discussion and conclusions

Brucellosis remains a common affliction in endemic areas worldwide [7]. The disease has not been effectively controlled, especially in developing countries, and its incidence has increased in recent years. Brucellar arthritis is caused by the spread of *B. melitensis* through blood to the hip joints and other large joints [8]. The incidence of brucellar arthritis among patients with osteoarticular brucellosis ranges from 18 to 60% [9]. In spite of its seriousness, this condition has not attracted enough interest among researchers. Hip arthritis is a serious complication of brucellosis and mainly characterized by hip pain and limited mobility. In the present report, both patients presented with marked hip pain and limited mobility. In addition, their ESR and CRP level were elevated. Septic hip arthritis is characterized by joint effusion, synovitis, and soft-tissue swelling on MR images. We believe that delay in diagnosis and treatment of any joint involvement might cause complications in the involved joint.

Brucellar hip arthritis is difficult to diagnose mainly because of its rare occurrence and non-specific clinical symptoms as well as the possibility of negative laboratory results in the chronic stage of the disease [6]. Therefore, in case of patients from endemic areas presenting with a complaint of non-specific and chronic joint pain, clinicians should have a high degree of suspicion of brucella infection.

With regard to diagnosis of brucellar hip arthritis, some authors suggest that the incubation time for bacterial cultures from synovial fluid should be extended to at least 3–4 weeks in order to increase the diagnostic rate [5]. PCR is a useful method for both initial diagnosis and detection of chronic brucellosis [10]. Brucellosis should be diagnosed by bacterial culture and PCR analysis of peripheral blood specimens or synovial fluid [11]. However, PCR findings are not always positive in case of focal brucellosis infections. At the same time, blood culture results are often negative in the subacute or chronic stage of the disease. In contrast, serological analysis is effective for etiological diagnosis of brucellosis. In fact, serological tests for brucella very rarely produce negative results in patients with focal brucellosis and, especially, osteoarthritis.

In case of relapse, patients should be treated with a standard regimen of drugs. The standard antibiotic regimen is doxycycline in combination with streptomycin or rifampicin for 6 weeks [12]. A triple treatment plan is necessarily advocated for septic arthritis such as TMP-SMX-Containing Regimens [13]. Complex complications of brucellosis, such as brucellar hip arthritis, require a longer treatment duration of not less than 3 months.

In conclusion, brucellar hip arthritis is a serious clinical manifestation of brucellosis, presenting mainly as marked joint pain and limited mobility. It is characterized by joint effusion, synovitis, and soft-tissue swelling on MR images. However, the condition might be difficult to diagnose because of its non-specific clinical and laboratory findings. Brucellosis should be diagnosed on the basis of bacterial culture and PCR findings. Physicians should consider brucellosis as one of the differential diagnoses of arthritis. Furthermore, brucellosis is an occupational disease, and slaughterhouse workers should be considered as being at a high risk of acquiring this zoonosis.

## Abbreviations

CRP: C-reactive protein
CT: Computed tomography
ESR: Erythrocyte sedimentation rate
MRI: Magnetic resonance imaging
PCR: Polymerase chain reaction
TAT: Tube agglutination test
